# Additively Manufactured Implants for Mandibular Overdentures: RCT Part 1 - Clinical and Peri-Implant Outcomes

**DOI:** 10.1590/0103-644020266875

**Published:** 2026-10-19

**Authors:** Lucas Jardim da Silva, Laura Lourenço Morel, Anna Paula da Rosa Possebon, Luciana de Rezende Pinto, Altair Anoninha Del Bel Cury, Jamil Awad Shibli, Otacílio Luiz Chagas Júnior, Fernanda Faot

**Affiliations:** 1 Graduate Program in Dentistry, School of Dentistry, Federal University of Pelotas (UFPel), Pelotas, RS, Brazil; 2 Department of Restorative Dentistry, School of Dentistry, Federal University of Pelotas (UFPel), Pelotas, RS, Brazil; 3 Dental Research Division, Guarulhos University, Guarulhos, São Paulo, Brazil; 4 Department of Oral and Maxillofacial Surgery and Maxillofacial Prosthodontics, School of Dentistry, Federal University of Pelotas, Pelotas, Rio Grande do Sul, Brazil; 5 Faculdade Israelita de Ciências da Saúde Albert Einstein, Hospital Israelita Albert Einstein, São Paulo, Brazil; 6 Department of Oral Medicine, Infection, and Immunity, Harvard School of Dental Medicine, Boston, Massachusetts, USA; 7 Department of Oral Health Sciences, Periodontology, KU Leuven & Dentistry, University Hospitals Leuven, Leuven, Belgium; 8 Department of Prosthodontics and Periodontology, Piracicaba Dental School, State University of Campinas (UNICAMP), Piracicaba, SP, Brazil

**Keywords:** Overdenture, Immediate Loading, Conventional Loading, Dental Implant, Additive Manufacturing

## Abstract

This study reported preliminary clinical outcomes of a randomized clinical trial comparing immediate and conventional loading protocols for mandibular overdentures supported by two narrow-diameter implants with a regular platform manufactured by additive technology. The focus was on primary implant stability, assessed using insertion torque and the implant stability quotient (ISQ). Sixteen completely edentulous participants (11 men, 5 women; mean age: 67.7 ± 7.6 years) received new complete dentures prior to surgery and were followed for up to 180 days, depending on the outcome assessed. Insertion torque was recorded at implant placement (motor and manual wrench), implant stability was measured at baseline and 90 days, and peri-implant healing and health parameters were evaluated at 7, 14, 30, 60, 90, and 180 days. A significance level of 5% was adopted for all statistical analyses. Immediate loading was performed on the day of implant placement, while conventional loading occurred after a 12-week healing period. The immediate loading group showed higher insertion torque (motor: 58.13 ± 5.18 Ncm, p=0.001; wrench: 58.44 ± 4.37 Ncm, p=0.004). ISQ increased significantly from baseline to 90 days in both groups (Immediate: 68.05 ± 6.08 to 72.44 ± 3.38; Conventional: 65.58 ± 5.47 to 70.72 ± 4.63; p<0.001), with no significant between-group differences (p=0.117). Peri-implant healing was more stable in the conventional group, with 100% of bone beds healed at 30 days (p=0.01). Significant between-group differences were observed at selected time points for peri-implant outcomes (p<0.05). Within the limitations of this preliminary short-term evaluation, both protocols appeared clinically feasible.

## Resumo

Este estudo relatou desfechos clínicos preliminares de um ensaio clínico randomizado que comparou os protocolos de carga imediata e convencional para overdentures mandibulares suportadas por dois implantes de diâmetro reduzido com plataforma regular, fabricados por manufatura aditiva. O foco foi a estabilidade primária dos implantes, avaliada pelo torque de inserção e pelo quociente de estabilidade do implante (ISQ).

Dezesseis participantes totalmente edêntulos (11 homens, 5 mulheres; idade média: 67,7 ± 7,6 anos) receberam novas próteses totais antes da cirurgia e foram acompanhados por até 180 dias, de acordo com o desfecho avaliado. O torque de inserção foi registrado no momento da instalação dos implantes (motor e torquímetro manual), a estabilidade dos implantes foi medida no baseline e aos 90 dias, e os parâmetros de cicatrização e saúde peri-implantar foram avaliados aos 7, 14, 30, 60, 90 e 180 dias. Um nível de significância de 5% foi adotado para todas as análises estatísticas. A carga imediata foi realizada no dia da instalação dos implantes, enquanto a carga convencional ocorreu após um período de cicatrização de 12 semanas.

O grupo de carga imediata apresentou maior torque de inserção (motor: 58,13 ± 5,18 Ncm, p=0,001; torquímetro: 58,44 ± 4,37 Ncm, p=0,004). O ISQ aumentou significativamente do baseline para 90 dias em ambos os grupos (Imediata: 68,05 ± 6,08 para 72,44 ± 3,38; Convencional: 65,58 ± 5,47 para 70,72 ± 4,63; p<0,001), sem diferenças significativas entre os protocolos (p=0,117). A cicatrização peri-implantar foi mais estável no grupo convencional, com 100% dos leitos ósseos cicatrizados aos 30 dias (p=0,01). Diferenças significativas entre os grupos foram observadas em momentos específicos para desfechos peri-implantares (p<0,05). Dentro das limitações desta avaliação preliminar de curto prazo, ambos os protocolos se mostraram clinicamente viáveis.

**Figure:**
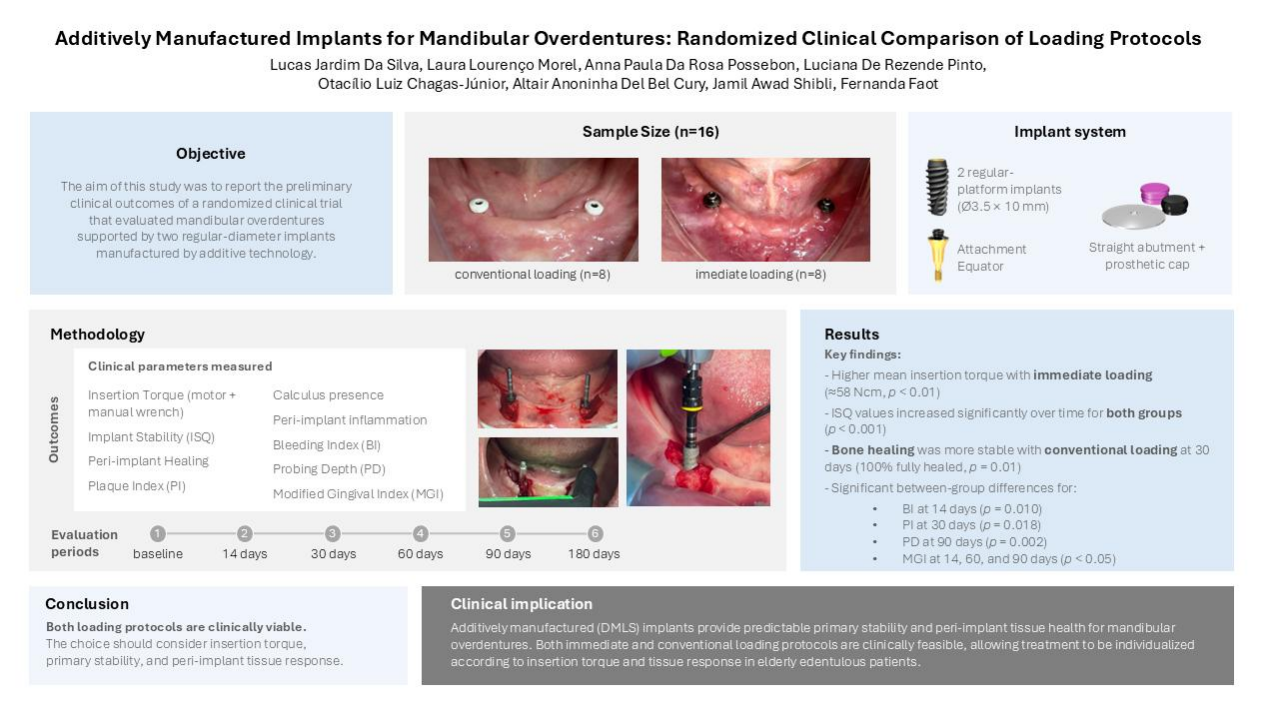

## Introduction

Additively manufactured implants, produced using the Direct Metal Laser Sintering (DMLS) technique, offer significant advantages in their surface characteristics. They stand out as a promising option, particularly for treating elderly patients who require enhanced bone integration and a reduced risk of biological complications, such as peri-implantitis. The DMLS technique enables the creation of complex topographies, with irregular peaks and valleys, which increase the contact area between the implant and bone tissue, thereby promoting osseointegration ^1^ ^,^ ^2^. In addition, rough and porous surfaces stimulate osteoblast adhesion, facilitating bone formation and improving both primary fixation and long-term stability ^3^. Studies such as that of Pingueiro et al. (2019) ^3^ demonstrate that DMLS-produced surfaces significantly reduce the adhesion of pathogens such as *Porphyromonas gingivalis* (red complex), one of the main bacteria associated with peri-implantitis. Another important advantage is that additive manufacturing eliminates the need for post-processing, allowing the production of textured surfaces directly during fabrication, while also enabling the development of bioactive surfaces with antimicrobial coatings that aid healing and reduce bacterial colonization ^1^ ^,^ ^2^ ^,^ ^3^. However, the potential benefits and drawbacks of these implants in elderly patients with full-arch rehabilitations remain unclear, since these patients present numerous physiological conditions that distinguish them from younger individuals ^4^.

Moreover, elderly individuals are often more susceptible to peri-implant diseases. According to Gündoğar et al. (2021) ^5^, patients aged 65 years and older have a greater propensity to develop peri-implantitis. This increased risk may be attributed to factors such as poor oral hygiene resulting from reduced dexterity, impaired vision, and immunosenescence.^6^ Additionally, with the gradual loss of peri-implant bone over time, the rough surface may become problematic for hygiene in patients with age-related manual dexterity limitations. ^4^ Therefore, focusing on elderly edentulous patients is clinically relevant when evaluating early peri-implant tissue response and maintenance needs during the healing phase.

In this context, these patients also present chronic residual ridge resorption as the main intraoral complication related to edentulism, which, over the years, leads to maxillary bone atrophy.^7^ This condition compromises the retention and stability of dentures, particularly mandibular ones, making their use and adherence to treatment more difficult ^8^. Treatment options include complete dentures, implant-retained prostheses, and implant-supported prostheses ^9^ ^,^ ^10^.

The choice between immediate loading and conventional loading of dental implants directly influences the biological processes involved in bone and tissue healing. Conventional loading, characterized by a waiting period of several weeks to months after implant placement, favors the initial formation of a stable and predictable bone-implant interface, minimizing micromovements that could compromise osseointegration. In contrast, immediate loading is commonly defined as prosthetic activation shortly after surgery (often within the first week) ^11^. Early loading differs from immediate loading by occurring after the immediate postoperative period but still within the first weeks to approximately 2 months, depending on the adopted consensus and definitions ^11^. This clarification is important because both terms are sometimes used interchangeably, which may influence the interpretation of clinical protocols and outcomes. Under appropriate conditions, immediate loading may reduce treatment time and improve patient acceptance, although it depends on strict criteria to ensure adequate primary stability ^11^. Evidence suggests that, under ideal conditions, immediate loading may achieve success rates comparable to conventional loading, especially in the anterior mandible, where bone quality is more favorable ^12^. However, factors such as bone density, surgical bed characteristics, insertion torque, and implant macrogeometry influence healing response, being even more critical in elderly populations or those with reduced tissue regeneration capacity ^13^.

Furthermore, recent studies have compared immediate and conventional loading protocols for mandibular overdentures supported by regular-diameter implants. Although meta-analyses have not identified significant differences in implant failure rates or marginal bone loss between the two protocols, whether overdentures were retained by two, three, or four implants ^14^ ^,^ ^15^, patient reports indicated a trend toward earlier improvements in oral health-related quality of life with immediate loading ^16^. However, clinical outcomes were inconsistent. While immediately loaded implants demonstrated more favorable probing depth values, they also showed higher scores for peri-implant inflammation and bleeding on probing ^17^. In addition, evidence from the literature suggests that peri-implant tissue indices, implant stability, and quality-of-life outcomes may not differ between the two loading protocols ^15^. Overall, both loading protocols appear to be viable options for mandibular overdentures, each with specific advantages and considerations during the healing phase.

Considering the scarcity of scientific evidence regarding the use of narrow-diameter implants with regular platforms produced by additive manufacturing, particularly in elderly patients, this article presents Part 1 of a randomized clinical trial (RCT). In this first stage, the study reports the preliminary results focused on the clinical performance and peri-implant tissue response of mandibular overdentures supported by two implants. A subsequent publication (Part 2) will address the functional outcomes and patient-reported quality of life, thus providing a broader understanding of the clinical applicability of this treatment approach.

This study aimed to compare immediate and conventional loading protocols for mandibular overdentures supported by two additively manufactured implants, focusing on primary implant stability, peri-implant healing, and peri-implant health parameters during short-term follow-up. The hypothesis was that, when adequate primary stability is achieved, immediate and conventional loading would present comparable primary implant stability, assessed by insertion torque and implant stability quotient (ISQ), with no relevant short-term differences in secondary peri-implant clinical outcomes between protocols.

## Materials and Methods

### Study design

This preliminary study is part of a randomized, parallel, prospective clinical trial, conducted in accordance with the Consolidated Standards of Reporting Trials (CONSORT) guidelines for reporting randomized controlled trials ^18^. The study was carried out in compliance with the Declaration of Helsinki (2008). The study protocol was approved by the institution’s Human Research Ethics Committee (protocol 6.420.934). The trial was registered in the Brazilian Clinical Trials Registry (ReBEC) under registration number RBR-9d94bxv.

In this preliminary phase, participants were followed for up to 180 days, with outcomes assessed at different time points depending on the endpoint (insertion torque at surgery; ISQ at baseline and 90 days; peri-implant clinical indices at 7, 14, 30, 60, 90, and 180 days). The present report focuses on early clinical and peri-implant outcomes. No formal sample size calculation was performed, as this investigation was designed as an exploratory/preliminary phase of the ongoing trial. The primary implant stability was considered the primary outcome and was assessed by insertion torque and implant stability quotient (ISQ). Surgical flap healing and peri-implant health parameters were considered secondary outcomes.

All participants were completely edentulous and received new bimaxillary complete dentures prior to implant placement. Patients were rehabilitated with two narrow-diameter implants with a regular platform (Ø3.5 × 10 mm, Regular, Plenum Bioengenharia, São Paulo, Brazil). The study protocol was approved by the institution’s Human Research Ethics Committee (protocol 6.420.934), and treatments were performed at the Complete Denture Clinic of UFPel. 

Inclusion criteria comprised fully edentulous patients who had been wearing conventional complete dentures in both arches for at least 3 months, controlled diabetics and hypertensive patients under medication, and those with sufficient bone availability without the need for bone grafting. Exclusion criteria included smokers, patients with severe or uncontrolled diabetes, blood disorders (such as hemorrhagic diathesis and anticoagulant use), severe systemic diseases (such as rheumatoid arthritis and osteogenesis imperfecta), immunosuppression (such as HIV or the use of immunosuppressive drugs), history of head or neck radiotherapy, previous oral implant placement, bisphosphonate use in the last 12 months, and, in the case of women, pregnancy or breastfeeding. Patients who constantly used or had a history of chronic use of analgesics, anti-inflammatory drugs, or psychotropic medications, as well as those requiring bone grafting at the implant site, were also excluded.

Cone-beam computed tomography (CBCT) (Eagle Edge 0.2 FS, Dabi Atlante) was performed to evaluate mandibular bone availability. Based on the images, the bone bed was previously mapped using Blue Sky Plan software (Libertyville, IL, USA), allowing analysis of bone dimensions and three-dimensional positioning of the implants. Bone quality was categorized according to the classification system used in this study (Lekholm and Zarb) ^19^, and bone morphology/crest characteristics were recorded during planning and surgery ^19^. Bone bed characterization was performed both before and after implant placement, with quantitative outcomes organized into specific tables for each experimental group (Figure 1).

**Figure 1:**
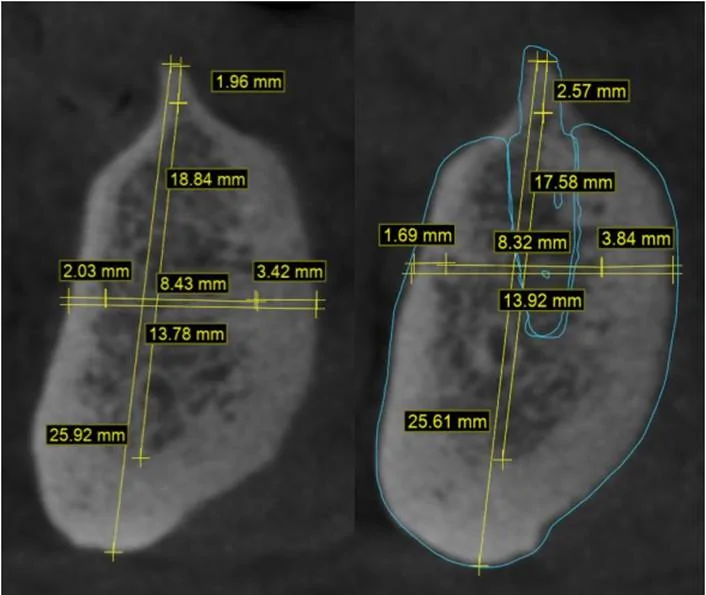
Bone bed characterization based on linear measurements in Blue Sky Plan. Linear measurements were performed in Blue Sky Plan to characterize the implant sites by assessing medullary and cortical bone. (a) Preoperative bone bed characterization performed in the pre-surgical CBCT within Blue Sky Plan. (b) Bone bed characterization performed on the preoperative CBCT using the exact implant position (blue) determined after segmentation of the postoperative CBCT and accurate alignment (blue) between the pre- and postoperative scans.

Bone quality at each implant site was classified according to the Lekholm and Zarb ^19^. Type I consists almost entirely of dense cortical bone; Type II presents a thick layer of cortical bone surrounding a core of dense trabecular bone; Type III presents a thin layer of cortical bone surrounding a core of trabecular bone of good strength; and Type IV presents a very thin cortical layer with low-density trabecular bone of poor strength. When applicable, an intermediate category (Type I-II) was used to describe sites presenting characteristics between Types I and II.

For randomization, patients were allocated in a 1:1 ratio to the Conventional Loading and Immediate Loading groups. Allocation details (conventional and immediate) were kept in sealed opaque envelopes to ensure allocation concealment and were disclosed to the surgeon and clinical team only during surgery, after implant placement and assessment of primary stability, at the time of prosthetic abutment installation. All participants received the intervention as originally allocated, and no crossovers occurred between groups. In the conventional loading group, prosthetic loading was performed after 12 weeks of healing, whereas in the immediate loading group, loading was performed on the same day as surgery (prosthetic activation on the day of implant placement), in accordance with consensus definitions and clinical recommendations for loading protocols ^11^. Because the allocated intervention could be clinically identified by the installed component (healing abutment in the conventional loading group and Equator attachment in the immediate loading group), blinding of follow-up clinical assessments was not feasible.

### Surgical procedure

A standardized one-stage surgical protocol was used by an experienced surgeon (OLCJ) to place all implants. Initially, intraoral and extraoral antisepsis was carried out with 0.12% and 2% chlorhexidine, respectively. The patient was then anesthetized in the vestibular and lingual sulcus with 4% Articaine 1:100,000 epinephrine (DFL Indústria e Comércio S/A). Partial-thickness L-shaped flaps with a mesial angle, without crossing the midline, were made at the preplanned sites in each case to expose the mandibular alveolar crest and identify the mental foramen (Figure 2). Osteotomies were guided by the position of the distal surface of the maxillary lateral incisors, approximately 5 mm anterior to the mental foramina, with a minimum inter-implant distance of 20 mm. A stepped drill sequence with under-preparation in diameter was used to place two narrow-diameter implants manufactured by additive technology (Ø3.5 × 10 mm Regular, Plenum Bioengenharia, São Paulo, Brazil). The sequence started with a Ø2.0 lance drill, followed by Ø2.15, Ø2.85, and Ø3.5 TAP drills, and implant placement using a contra-angle driver (Figure 3). When sharp/irregular crests were identified and could compromise flap adaptation, denture comfort, or the creation of a stable implant platform, minor bone regularization (alveoloplasty/osteoplasty) was performed to obtain a smoother, flatter crest before implant placement.

**Figure 2:**
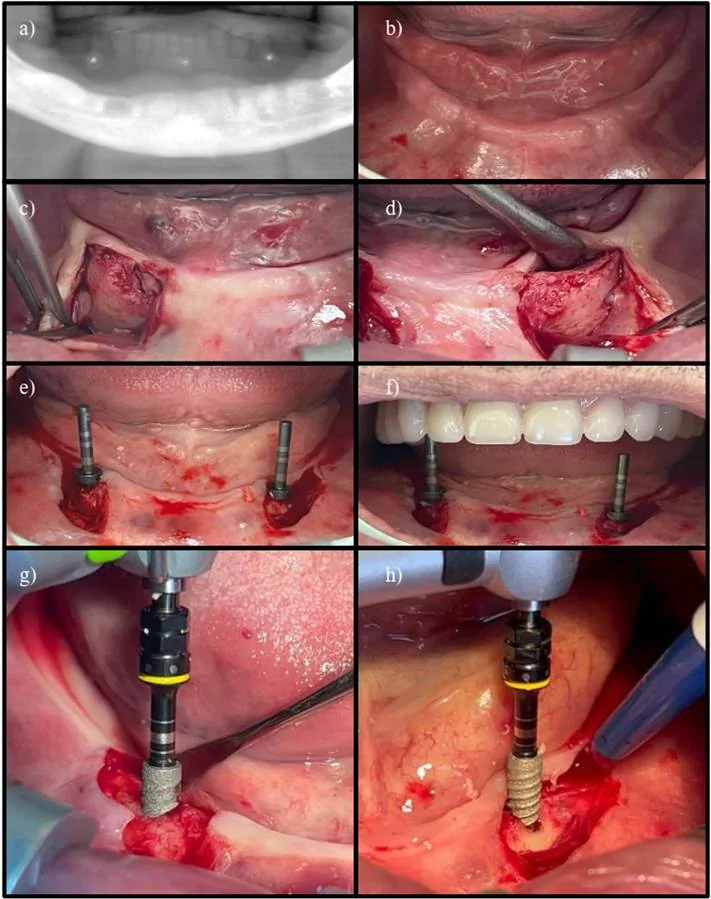
Surgical sequence showing the Modified Neumann Incision a) pre-operatory x-ray; b) Initial residual ridge; c) L-shaped incision; d) elevation of the mucoperiosteal flap; e) parallelometer positioned on the right side; e) parallelometer positioned on both right and left sides; f) frontal view of the parallelometers indicating the correct mesiodistal positioning of the drilling; g) left implant installation; right implant installation.

**Figure 3:**
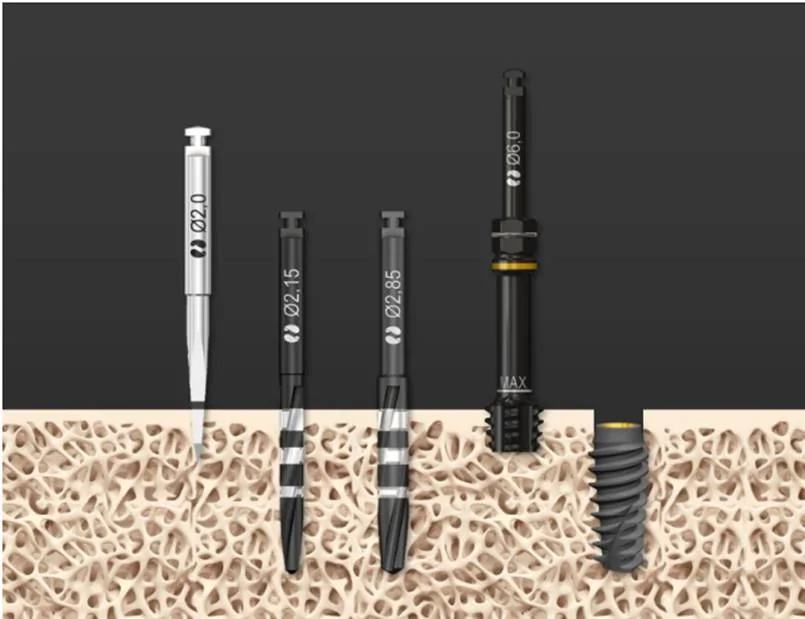
Drill sequence for the placement of 3.5 × 10 mm implants used in the study.

### Primary stability (IT and ISQ) measurement

During implant placement, insertion torque (IT) was recorded using the surgical motor and the surgical wrench, both with a fixed calibration of 60 Ncm. A minimum IT threshold of 32 Ncm was adopted as a criterion to support immediate loading predictability ^20^. After implant placement and IT assessment, allocation was disclosed by opening the sealed envelope. If the IT threshold for immediate loading had not been achieved by a participant allocated to the immediate loading group, the participant would have been managed under the conventional loading protocol for safety reasons; however, this protocol deviation did not occur, and no participants required reassignment, as all individuals allocated to immediate loading achieved IT ≥ 32 Ncm.

A Type 45 SmartPeg was connected to the implants to measure the implant stability quotient (ISQ) in four directions (mesial, distal, buccal, and lingual) using the Osstell® device. Finally, either a healing abutment (conventional loading) or the Equator attachment (Plenum Bioengenharia, Jundiaí, SP, Brazil) (immediate loading) was connected to the implant, and the mucoperiosteal flap was adapted and sutured with simple interrupted stitches to ensure that the components remained exposed rather than submerged (Figure 4). 

**Figure 4:**
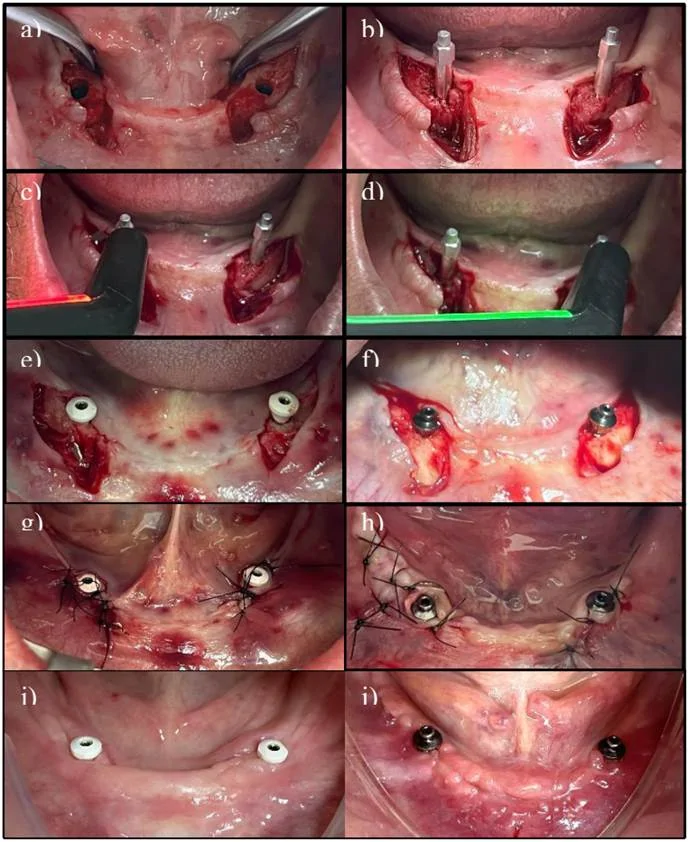
ISQ measurement with the Osstell® device. a) Implants installed; b) installation of SmartPeg Type 45 on the implants; c) ISQ measurement with the Osstell® device on the right implant; d) ISQ measurement with the Osstell® device on the left implant; e) installation of a PEEK healing abutment for the conventional-loading group; f) installation of the Equator attachment for the immediate-loading group; g) immediate postoperative view — conventional-loading group; h) immediate postoperative view — immediate-loading group; i) 14-day postoperative view — conventional-loading group; j) 14-day postoperative view — immediate-loading group.

### Prosthetic procedures 

All participants received new complete dentures prior to implant surgery. In the conventional loading group, the new mandibular complete denture was relined using a temporary soft liner (Soft Provisório, TDV Dental, Brazil), which was replaced monthly during the 12-week healing period. After completion of the healing period (12 weeks), the mandibular denture was converted to an overdenture by connecting the Equator attachment system (Plenum Bioengenharia, Jundiaí, SP, Brazil) and performing the pickup procedure using the same acrylic resin protocol described for the immediate loading group. In the immediate loading group, mandibular overdentures were loaded on the day of surgery by an experienced prosthodontist (LJS). A straight cap was connected for intraoral fixation of the Equator attachment (Plenum Bioengenharia, Jundiaí, SP, Brazil) using self-curing acrylic resin for prostheses (JET®, Clássico - Artigos Odontológicos, São Paulo, Brazil), adapting the system to the internal surface of the mandibular denture (Figure 5).

**Figure 5:**
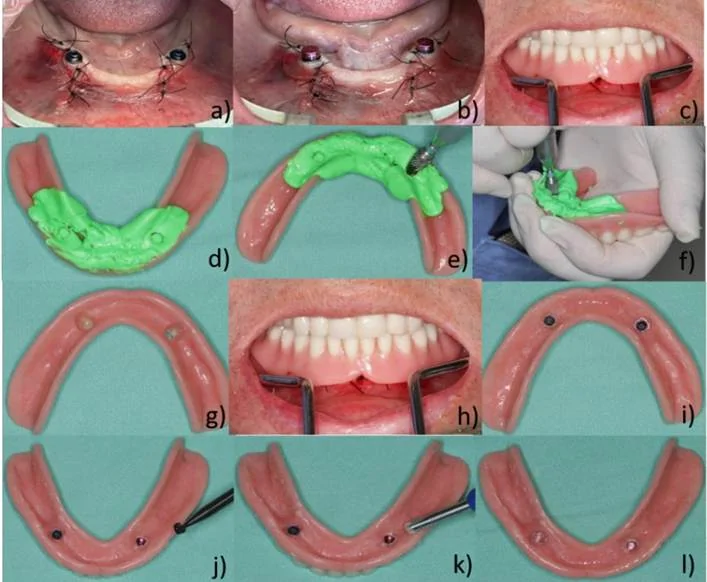
Illustration of the transition from CD to IMO according to the manufacturer instruction: a) Immediate postoperative view with Equator placement; b) Placement of straight caps on the Equators; c) Application of light-body addition silicone to the anterior region of the conventional complete denture and positioning of the patient in occlusion to mark the areas that need to be relieved in the denture for the pickup procedure; d) Addition silicone showing the areas to be relieved; e) Relief of the marked areas using a straight handpiece and burs; f) Additional relief of the marked areas with a straight handpiece and burs; g) Relief of the areas that will receive the straight caps, which are then filled with pink acrylic resin; h) The denture is positioned in occlusion and maintained for 5 minutes; i) Straight caps fixed to the denture after pickup and removal of excess acrylic resin; j) Removal of the prosthetic coping; k) Placement of the standard Plenum coping; l) Final appearance of the overdenture.

### Postoperative examinations, care, and medications

After surgery, patients were instructed to undergo panoramic radiography for monitoring marginal bone loss and to follow the prescribed medication correctly. Additional recommendations included: avoid spitting, blowing the nose, or sneezing with the mouth closed; maintain a liquid or soft cold diet for the first 3 days; gradually increase food consistency from the fourth day onward; refrain from physical exertion until suture removal; avoid sun exposure for 15 days; avoid drinking hot liquids for 7 days; avoid rinsing the mouth for 15 days; brush teeth normally but carefully in the surgical area (without rinsing); remain seated, and when sleeping use a slightly higher pillow than usual; avoid excessive talking; and avoid passing the tongue over the surgical wound. For patients who received immediate loading, additional instructions included not removing the prosthesis during the first 48 hours to help control edema and performing oral hygiene with a 20 mL syringe and saline solution under the overdenture. Preoperative medication included 40 mg prednisolone administered 1 hour before the procedure. Postoperative medication included amoxicillin 500 mg three times daily for 7 days, ibuprofen 600 mg three times daily for 4 days, and paracetamol 500 mg four times daily for 3 days. Nylon sutures (Procare®; Lamedid Ltda, Barueri-SP, Brazil) were removed 10 days after surgery. Complete postoperative care instructions and prosthesis care guidelines were provided to all patients and reinforced at each follow-up appointment.

### Implant stability

Primary implant stability was assessed using resonance frequency analysis (Osstell®-Integration Diagnostics AB, Gothenburg, Sweden). A SmartPeg™ Type 45 was manually connected to the internal connection of the implants, and the Osstell probe was positioned perpendicular to the SmartPeg™. ISQ values were measured in four directions (mesial, distal, buccal, and lingual) at implant placement (baseline) and at 90 days postoperatively. All measurements were performed in triplicate by the same examiner (LJS).

### Clinical monitoring and data collection

Clinical examination of the implants was performed at four sites per implant (mesial, distal, buccal, and lingual), following the protocol of a previous study by Bielemann et al. (2018) ^17^. A single experienced specialist (LJS) evaluated the clinical parameters of the implant sites, including visual plaque index (VP), calculus presence (CP), peri-implant inflammation (PI), probing depth (PD), and bleeding on probing (BOP). In addition, surgical flap healing (SFH) was assessed at each follow-up visit by direct visual inspection of the surgical wound using an ordinal clinical healing score. The definitions of each clinical parameter and the procedures used for their assessment are provided in Supplementary Table S1.

SFH, VP, CP, and PI were evaluated at 1, 2, 4, 8, 12, and 24 weeks. PD and BOP were measured at 2, 4, 8, 12, and 24 weeks after surgery.

### Data analysis

A descriptive analysis was performed. Normality was assessed using the Shapiro-Wilk and Kolmogorov-Smirnov tests, and homogeneity of variances was assessed using Levene’s test. ISQ and probing depth met parametric assumptions; therefore, ISQ was analyzed using a two-factor repeated-measures ANOVA (group × time: baseline and 90 days). PD changes over time within each loading protocol were analyzed using the Friedman test followed by paired Wilcoxon post hoc tests. Insertion torque (motor and wrench) and peri-implant soft-tissue indices did not meet parametric assumptions and were analyzed using non-parametric tests: Mann-Whitney for between-group comparisons of insertion torque, and Chi-square tests for between-group comparisons of categorical peri-implant outcomes, with McNemar tests for within-group paired comparisons across time points. In this preliminary phase, the patient was considered the unit of analysis. Although two implants were placed in each participant, outcomes were analyzed longitudinally at the patient level across the different follow-up periods, and implant-level observations were not treated as independent units for statistical inference. Given the preliminary/exploratory nature of this phase and the multiple comparisons across outcomes and time points, no formal multiplicity adjustment was applied; findings should be interpreted accordingly. All participants were analyzed according to their original randomized allocation (intention-to-treat). As no protocol deviations/crossovers occurred, intention-to-treat and per-protocol analyses were equivalent for the outcomes reported in this preliminary phase. SFH were categorized into three groups: 0 for complete repair (E1), 1 for the presence of fibrin (E2 + E3), and 2 for the presence of necrosis (E4 + E5). VP, CP, PI, and BOP were dichotomized as 0 (absence) and 1 (presence). This categorization (E1-E5 condensed into 0-2) was adopted to improve clinical interpretability and to allow stable categorical comparisons in a small, exploratory sample across repeated time points.

## Results

Figure 6 presents the CONSORT flow diagram of participant screening, randomization, allocation, follow-up, and analysis. Twenty-three patients were assessed for eligibility. Seven were excluded before randomization, and 16 participants were randomized and allocated to either the immediate loading group (n = 8) or the conventional loading group (n = 8). All randomized participants received the allocated intervention, completed follow-up, and were included in the analysis.

**Figure 6:**
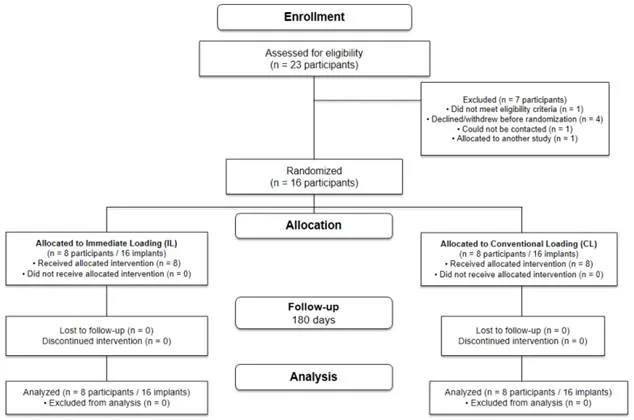
CONSORT flow diagram showing participant screening, randomization, allocation, follow-up, and analysis. IL, immediate loading; CL, conventional loading.

### Sample characterization

The sample consisted of 16 participants, including five women and eleven men, with an overall mean age of 67.69 ± 7.58 years. The mean age was 61.88 ± 9.98 years in the Immediate Loading group and 73.50 ± 5.19 years in the Conventional Loading group (Table 1). The mean time of mandibular edentulism was 8.19 ± 9.32 years. Most patients did not present mandibular atrophy (13/16). Site-related baseline characteristics were recorded for the 32 implant sites (two implants per participant; 16 sites per group), as shown in Table 1. Regarding ridge morphology, the “knife-edge” pattern was predominant (n=8), followed by normal (n=3), plateau (n=3), and retentive/undercut (n=2). According to the bone quality classification adopted in this study (Lekholm and Zarb classification; described in the Methods section),^19^ site distributions included Type I, Type I-II, Type II, and Type III categories (Table 1). Bone regularization was required in 23 sites, while nine sites did not require this procedure (Table 1). Regularization consisted of minor crestal regularization (alveoloplasty/osteoplasty) to obtain a smoother and flatter crest when sharp or irregular ridge anatomy could compromise flap adaptation and/or prosthesis comfort. Concerning bone level, five sites were classified as bone level, 11 as Sub1, and 16 as Sub2.

The overall mean bone density was 577.47 ± 203.55 HU and was higher in the immediate loading group (695.5 ± 213.65 HU) compared with the conventional loading group (459.44 ± 193.46 HU) (Table 1). This baseline imbalance should be considered when interpreting between-group comparisons, as bone density may influence primary stability and early peri-implant outcomes.

**Table 1:** Sample characterization. IL, Immediate Loading; CL, Conventional Loading

	IL (n=8)	CL (n=8)	Overall
Gender (Women/Men)	2/6	3/5	5/11
Age (years)	61.88 ± 9.98	73.50 ± 5.19	67.69 ± 7.58
Time of mandibular edentulism (years)	7.00 ± 8.25	9.38 ± 10.39	8.19 ± 9.32
Mandibular Atrophy (No/Yes)	7/1	6/2	13/3
Bone shape (Knife-edge/Normal/Undercut/Plateau)	4/1/2/1	4/2/0/2	8/3/2/3
Bone type (Type I/Type I-II/Type II/Type III)	7/5/4/0	1/8/5/2	8/13/9/2
Bone regularization (No/Yes)	4/12	5/11	9/23
Bone level (Level/Sub 1mm/Sub 2mm)	3/3/10	2/8/6	5/11/16
Density	695.5 ± 213.65	459.44 ± 193.46	577.47 ± 203.55

### Insertion torque

Insertion torque (IT) values are summarized in Table 2. The Immediate Loading group presented higher insertion torque values measured by both the motor and the manual wrench (motor: 58.13 ± 5.18 Ncm; wrench: 58.44 ± 4.37 Ncm) compared with the Conventional Loading group (motor: 42.31 ± 12.98 Ncm; wrench: 40.63 ± 16.78 Ncm). Between-group differences were statistically significant for both motor IT (p=0.001) and wrench IT (p=0.004) (Mann-Whitney test). These differences were expected because immediate loading required meeting the predefined primary stability criterion (IT ≥ 32 Ncm). Importantly, all participants allocated to immediate loading achieved the IT threshold, and no protocol deviations or crossovers occurred.

**Table 2:** Mean, standard deviation, minimum, and maximum values, and significance level of insertion torque measured by motor and manual wrench.

	Motor	Manual Wrench
Immediate	58.13 ± 5.18 (43 - 60)	58.44 ± 4.37 (45 - 60)
Conventional	42.31 ± 12.98 (21 - 60)	40.63 ± 16.78 (15 - 60)
p-value	0.001	0.004

### Implant Stability Quotient (ISQ)

The mean, standard deviation, minimum, and maximum values, as well as the significance level of ISQ, are presented in Table 3. 

**Table 3:** Mean, standard deviation, minimum and maximum values, and significance level of the Implant Stability Quotient (ISQ).

	Baseline mean (±SD)	90 days mean (±SD)	Mean (±SD)
Immediate	68.05 ± 6.08	72.44 ± 3.38	70.25 ± 4.73
	(54.50 -77.20)*	(66.00 - 78.25)*	(54.50 - 78.25)
Conventional	65.58 ± 5.47	70.72 ± 4.63	68.15 ± 5.05
	(52.50 - 73.50)*	(60.50 - 78.00)*	(52.50 - 78.00)

* Asterisks indicate a significant effect of time (p=0.000) observed within the groups from baseline to 90 days.

ISQ increased significantly over time in both groups (p<0.001). In the Immediate Loading group, mean ISQ increased from 68.05 ± 6.08 at baseline to 72.44 ± 3.38 at 90 days. In the Conventional Loading group, mean ISQ increased from 65.58 ± 5.47 at baseline to 70.72 ± 4.63 at 90 days. No statistically significant differences were observed between loading protocols (p=0.117), and no significant time-by-group interaction was detected (p=0.809), indicating that both protocols demonstrated a similar pattern of stability increase during early healing.

### Peri-implant Health Outcomes

Peri-implant health outcomes are summarized in Table 4 (between-group comparisons), and within-group comparisons are provided in Table S2 (Supplementary Material). Overall, both protocols showed progressive soft-tissue healing over the early postoperative period, with a reduction of fibrin/necrosis findings and stabilization thereafter. Bone bed healing was more stable in the Conventional Loading group, reaching complete healing at 30 days (p = 0.01).

For visual plaque index and calculus presence, the frequency of biofilm-related findings increased over time in both groups, with statistically significant between-group differences at selected time points (Table 4). Inflammatory indices (peri-implant inflammation and bleeding on probing) also changed over time and differed between groups at specific follow-ups (Table 4). Probing depth values decreased during follow-up in both protocols, suggesting progressive peri-implant tissue maturation.

### Complications, relining, and maintenance follow-ups 

No implant failures or major biological or mechanical complications were observed during the follow-up period. Maintenance follow-ups were performed according to the prespecified schedule (7, 14, 30, 60, 90, and 180 days) to monitor healing and reinforce hygiene instructions. In the Conventional Loading group, the mandibular complete denture was relined with a temporary soft liner as part of the protocol, which was replaced monthly during the 12-week healing period; routine minor adjustments and hygiene reinforcement were provided as needed at follow-up visits.

**Table 4:** Descriptive analysis of peri-implant health outcomes, including surgical flap healing (SFH), visual plaque (VP), calculus presence (CP), peri-implant inflammation (PI), bleeding on probing (BOP, %), and probing depth (PD, mean ± SD, mm).

Groups	Immediate Loading						Conventional Loading					
Outcomes/ Time (days)	7	14	30	60	90	180	7	14	30	60	90	180
*SFH*												
Without Fibrin (%)	10 (62.5)	14 (87.5)	16 (100)	16 (100)	16 (100)	0 (0.00)	4 (28.58)	14 (100)	14 (100)	14 (100)	14 (100)	-
With Fibrin (%)	2 (12.5)	0 (0.00)	0 (0.00)	0 (0.00)	0 (0.00)	7 (50.00)	7 (50.01)	0 (0.00)	0 (0.00)	0 (0.00)	0 (0.00)	-
Necrosis (%)	4 (25.00)	2 (12.5)	0 (0.00)	0 (0.00)	0 (0.00)	7 (50.00)	3 (21.43)	0 (0.00)	0 (0.00)	0 (0.00)	0 (0.00)	-
*VP Presence (%)*	6 (37.5)	14 (87.5)	12 (75)	13 (81.25)	14 (87.5%)	13 (81.2)	4 (28.58)	6 (42.86)	9 (64.29)	10 (71.42)	10 (71.42)	14 (100)
*CP (%)*	0 (0.00)	2 (12.5)	2 (12.5)	4 (25)	8 (50%)	3 (18.75)	0 (0.00)	2 (14.28)	3 (21.43)	2 (14.28)	0 (0.00)	3 (21.43)
*PI Presence (%)*	12 (75)	10 (62.5)	1 (6.25)	2 (12.5)	5 (31.25%)	6 (37.5)	10 (71.42)	12 (85.72)	6 (42.86)	3 (21.43)	0 (0.00)	0 (0.00)
*BOP Presence (%)*	-	10 (15.63)	11 (68.75)	11 (68.75)	12 (75%)	9 (56.25)	-	14 (100)	12 (85.72)	14 (100)	14 (100)	10 (71.42)
*PD (mm)*	-	2.49 ± 0.99	2.20 ± 1.04	2.06 ± 0.99	1.75 ± 0.82	1.83 ± 0.76	-	3.70 ± 0.98	2.74 ± 1.16	2.48 ± 1.10	2.11 ± 1.25	1.98 ± 1.13

Significant differences between groups are bolded and were observed for VP at 14 days (p=0.01), CP at 90 days (p=0.02), PI at 30 days (p=0.01), 90 days (p=0.02), and 180 days (p=0.01), and BOP at 14 days (p=0.01), 60 days (p=0.04), and 90 days (p=0.04) (Chi-square test).

## Discussion

The hypothesis of this preliminary trial was partially supported. Although both groups showed adequate primary implant stability and a significant increase in ISQ from baseline to 90 days, the immediate-loading group had higher insertion torque values than the conventional-loading group. Regarding the secondary outcomes, both protocols showed satisfactory soft tissue healing, with progressive regression of fibrin and necrosis over time, although selected peri-implant clinical parameters differed between groups at specific follow-up periods. These observations should be interpreted within the context of short-term follow-up and exploratory analyses.

Initially, insertion torque showed significantly higher values in the immediate loading group, which was expected due to the minimum torque threshold used as a clinical criterion to support immediate loading predictability ^20^. This finding is consistent with the concept that primary stability is a key prerequisite for successful early loading protocols, and it is influenced by bone quality, surgical technique, and implant macrogeometry ^21^ ^,^ ^24^. At the same time, excessively high insertion torque has been discussed as a potential contributor to increased mechanical stress at the bone-implant interface, which could influence early remodeling ^22^. Conversely, the conventional loading group presented lower torque values, and the short-term clinical performance was not compromised within the observation period, aligning with evidence that implants may still perform adequately under lower torque values, particularly when a healing period is allowed ^23^ ^,^ ^24^.

ISQ increased significantly over time in both groups, with no statistically significant differences between loading protocols at 90 days. This overall behavior is consistent with the expected pattern of increasing secondary stability during early osseointegration and with clinical trials and reviews that indicate comparable stability outcomes across different loading strategies under controlled conditions ^11^ ^,^ ^24^ ^,^ ^25^ Importantly, baseline bone density was higher in the immediate loading group, which may have favored primary stability and influenced early stability and peri-implant outcomes. Because bone density and site characteristics can affect primary stability and early healing responses, this baseline imbalance should be considered a potential confounder when interpreting between-group comparisons in this preliminary phase ^11^ ^,^ ^13^ ^,^ ^21^ ^,^ ^24^.

Regarding peri-implant health, the overall trend toward improvement in soft tissue healing over time is consistent with early postoperative recovery. However, between-group differences were observed at selected time points for plaque/calculus-related findings and inflammatory indices. These results reinforce that immediate loading may require stricter monitoring and reinforcement of hygiene and maintenance strategies during the healing phase, especially in older adults who may face limitations in plaque control ^5^ ^,^ ^6^. From a clinical perspective, current guidelines and systematic evidence emphasize that prevention and supportive care are central to minimizing peri-implant disease risk, highlighting the importance of periodic maintenance and patient-centered hygiene instruction ^29^ ^,^ ^30^ ^,^ ^31^ ^,^ ^32^. When interpreting statistically significant differences across multiple indices and time points, it is also important to consider the magnitude of the differences and whether they are clinically meaningful, rather than relying solely on p-values, particularly in exploratory analyses.

The short-term findings of this study fit within the broader evidence comparing immediate versus conventional loading for mandibular overdentures. Systematic reviews and meta-analyses generally indicate similar implant survival and marginal bone outcomes between protocols, while patient-reported outcomes may show earlier improvements with immediate loading in some scenarios ^14^ ^,^ ^15^ ^,^ ^16^. Nevertheless, clinical peri-implant indices can behave inconsistently across studies, with some trials reporting more favorable probing depth profiles but higher inflammatory markers under immediate loading, suggesting that protocol selection should consider both surgical stability criteria and peri-implant tissue response during follow-up ^15^ ^,^ ^17^ ^,^ ^33^.

With respect to additively manufactured implants, DMLS surfaces have been described as offering complex topography and porosity that may support early bone response and improve osseointegration dynamics ^1^ ^,^ ^2^ ^,^ ^3^ ^,^ ^27^ ^,^ ^28^. At the same time, statements regarding long-term microbiological behavior and peri-implant disease risk in porous surfaces should be interpreted with caution as hypotheses rather than definitive conclusions, especially given that microbiological findings are highly dependent on surface chemistry and topography, as well as patient-level hygiene/maintenance variables ^3^ ^,^ ^34^. Therefore, longer-term clinical studies are necessary to clarify whether the early advantages in bone response translate into sustained peri-implant health outcomes over time ^27^ ^,^ ^28^ ^,^ ^34^.

From a clinical standpoint, additively manufactured narrow-diameter implants with a regular platform may broaden treatment options for mandibular overdentures, including in patients with reduced ridge dimensions where more invasive interventions would otherwise be considered ^35^ ^,^ ^36^ ^,^ ^37^. However, immediate loading may be less favorable in situations where adequate primary stability cannot be achieved (e.g., insertion torque below the predefined threshold), in markedly compromised bone conditions, or in patients with limited ability to maintain hygiene and attend maintenance follow-ups-considerations that are particularly relevant in elderly populations ^5^ ^,^ ^6^ ^,^ ^20^ ^,^ ^21^.

This study has limitations that must be acknowledged. As a preliminary phase, the sample size was small, and no formal power calculation was performed, which limits the ability to detect differences between protocols. Another limitation is that blinding of follow-up clinical assessments was not feasible because the installed prosthetic components allowed identification of the allocated intervention. Additionally, follow-up was outcome-dependent (ISQ assessed up to 90 days, while peri-implant clinical indices were followed up to 180 days). The number of outcomes and time-point comparisons increases the risk of false-positive findings in the absence of multiplicity adjustment. The sample was heterogeneous, with an imbalanced gender distribution, and baseline differences between groups-particularly in bone density-may have influenced outcomes. Therefore, these findings should be considered exploratory and hypothesis-generating, and continuation of the trial with larger samples and longer follow-up is essential.

Taken together, these preliminary findings suggest that both immediate and conventional loading may be clinically applicable for mandibular overdentures supported by additively manufactured narrow-diameter implants when appropriate case selection and stability criteria are met. However, the exploratory nature of this phase, the small sample, baseline imbalance in bone density, and the short follow-up preclude definitive conclusions regarding superiority or long-term clinical advantage of either protocol.

## Conclusion

Within the limitations of this preliminary short-term evaluation, both immediate and conventional loading protocols were clinically feasible for mandibular overdentures supported by additively manufactured implants. Immediate loading was associated with higher insertion torque, whereas both protocols showed increased ISQ over time and overall improvement in peri-implant clinical conditions during follow-up. These findings should be interpreted cautiously and require confirmation in larger studies with longer follow-up.

## Data Availability

The research data are available upon request.
